# Lessons for aging from Werner syndrome epigenetics

**DOI:** 10.18632/aging.102829

**Published:** 2020-02-05

**Authors:** George M. Martin, Martin Poot, Thomas Haaf

**Affiliations:** 1 Department of Pathology, University of Washington, Seattle, WA 98195, USA; 2 Institute of Human Genetics, Julius-Maximilians University, 97074 Wuerzburg, Germany

**Keywords:** premature aging, developmental origins, epigenetic changes, transcriptional disease, Werner syndrome

The Werner syndrome (WRN) [1] is a canonical member of a family of genetically determined disorders that include multiple phenotypes consistent with their characterizations as segmental progeroid syndromes [2]. It is important to note, however, that these syndromes may include discordances with the usual phenotypic features of aging, hence the use of the suffix oid (i.e., *like* aging). For example, the ratio of epithelial to non-epithelial cancers in WRN is 1:1, whereas the ratio seen in usual aging is 10:1 [3]. Moreover, while WRN research has contributed to the widespread acceptance of genomic instability as one of the hallmarks of aging, features such as variegated translocation mosaicism and a preponderance of large deletions are particularly characteristic of WRN.

The purpose of this editorial, however, is to review and elaborate upon “epigenetic signatures of Werner syndrome occur early in life and are distinct from normal epigenetic aging processes” [4]. Although our studies did show that WRN and several other progeroid syndromes were epigenetically distinct disorders, given the comparatively large sample size of peripheral blood specimens from WRN and their age-matched controls, we shall focus our discussion upon those results. The first point to make is that, unlike the highly informative epigenetic clock studies, which utilized DNA methylation markers across several hundred highly selected CpG sites, we assessed >800,000 CpGs representing the entire epigenome. The vast majority (>90%) of differentially methylated CpGs and regions (DMRs) in WRN were not affected by aging, consistent with the view that WRN is not merely accelerated normal aging. A particularly striking finding was that DMRs were enriched in genes associated with transcription factor activity, leading us to hypothesize that WRN might best be conceptualized as a disease based upon aberrant controls of the expressions of a wide array of genetic loci, some of which are plausibly related to clinical phenotypes of WRN (Figure 1). Moreover, given that the methylation changes in the highest ranking DMR in the promoter region of the *HOXA4* gene as well as in other DMRs preceded disease manifestation, it seems likely that these transcriptional aberrations began early in development. That finding reinforces the concept that “how well one builds an organism makes a great deal of difference on how long it lasts and how well it functions!” [5].

**Figure 1:**
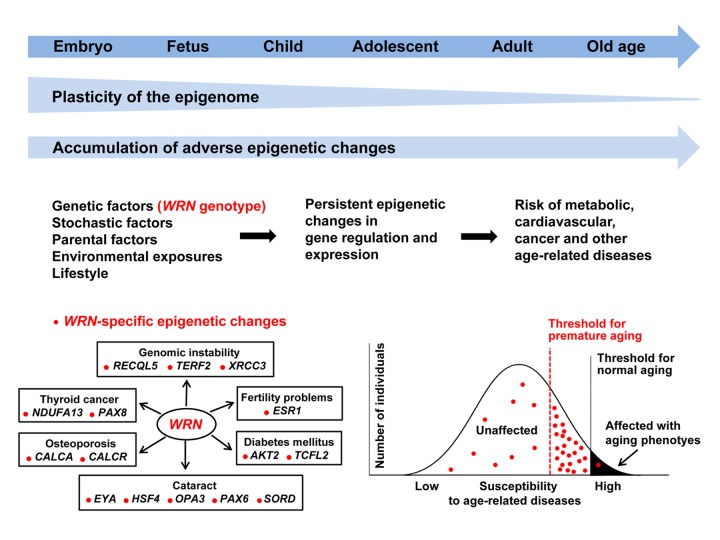
**Developmental origins of premature and normal aging.** Individuals with WRN are endowed with specific epigenetic changes in several hundred genes (indicated by red dots) before the disease manifests. This is consistent with a multifactorial model of aging, in which the WRN-specific epigenetic burden, one that may arise early in development, is associated with a lowered threshold and increased risk for age-related diseases. In order to be affected, the number of adverse factors including epigenetic changes (red dots) in an individual must exceed a certain threshold.

The epigenome of an individual is most plastic during early development and is shaped by a plethora of stochastic, internal (i.e. genetic variation), and external factors (i.e. environmental exposures). The epigenetic changes associated with WRN and other segmental progerias are widespread but are all of small effect size. Both premature and normal aging phenotypes may manifest when the adverse factors exceed a critical threshold. Individuals with WRN may be endowed with an epigenome(s) early in life which lowers their threshold for developing a number of specific aging phenotypes (Figure 1). This links methylation changes in WRN to research on intergenerational pathology. Moreover, it suggests novel areas of research such as the possible interrelationships between genomic instability, transcription, DNA methylation, and premature ageing in disorders such as Cockayne syndrome, trichothiodystrophy, and Xeroderma pigmentosum and to inherited longevity in the healthy population.

The important role of developmental biology in the shaping of our healthspans and lifespans is highlighted by research on the impacts of environmental factors upon epigenetically based intergenerational and transgenerational inheritance, research that had its origins with the infamous Dutch famine near the end of World War 2 (https://www.nytimes.com/2018/01/31/science/dutch-famine-genes.html). Although a recent review has critically discussed the transmission of epigenetic information through the mammalian germline and urged more highly controlled experiments [6], a growing number of animal and human studies raise great concerns about the long range public health significance of epigenetic forms of heredity [7].

## References

[1] Oshima J, et al. Ageing Res Rev. 2017; 33:105–14. 10.1016/j.arr.2016.03.002 26993153 PMC5025328

[2] Lessel D, Kubisch C. Dtsch Arztebl Int. 2019; 116:489–96. 10.3238/arztebl.2019.0489 31452499 PMC6726857

[3] Goto M, et al. Cancer Epidemiol Biomarkers Prev. 1996; 5:239–46. 8722214

[4] Maierhofer A, et al. Aging Cell. 2019; 18:e12995. 10.1111/acel.12995 31259468 PMC6718529

[5] Martin GM. Exp Gerontol. 2017; 94:46–51. 10.1016/j.exger.2016.11.008 27871822 PMC5438291

[6] Horsthemke B. Nat Commun. 2018; 9:2973. 10.1038/s41467-018-05445-5 30061690 PMC6065375

[7] Nilsson EE, et al. Environ Epigenet. 2018; 4:dvy016. 10.1093/eep/dvy016 30038800 PMC6051467

